# Efficacy of the Renal-guard system in the prevention of contrast-induced nephropathy following cardiac interventions among patients with chronic kidney disease

**DOI:** 10.3389/fcvm.2025.1438076

**Published:** 2025-03-03

**Authors:** Farah Yasmin, Yusra Mashkoor, Hala Najeeb, Ayra Asim Shaikh, Butool Nusrat, Abdul Moeed, Muhammad Sohaib Asghar, Chadi Alraies

**Affiliations:** ^1^ Dow University of Health Sciences, Karachi, Pakistan; ^2^ Ziauddin University, Karachi, Pakistan; ^3^ AdventHealth, Sebring, FL, United States; ^4^ Cardiovascular Institute, Detroit Medical Center, Detroit, MI, United States

**Keywords:** contrast-induced nephropathy, CKD, MACE, mortality, RenalGuard, meta-analysis

## Abstract

**Background:**

Contrast-induced nephropathy (CIN), also called as contrast associated-acute kidney injury (CA-AKI) is a common complication following cardiac procedures. KDIGO guidelines define CIN as a ≥25% increase in serum creatinine or an absolute increase of at least 0.5 mg/dl 48–72 h post-contrast administration. The single most effective measure in preventing CIN is peri-procedural intravascular hydration typically from 12 h before to 24 h after contrast media exposure but has limitations. Recently, the RenalGuard (RG) system has emerged as a new tool, demonstrating safer and more efficient hydration and reducing the incidence of AKI caused by CIN.

**Aims:**

We conducted this meta-analysis on the effectiveness of the RG system in preventing CIN in patients undergoing cardiac interventions.

**Methods:**

A comprehensive literature search of PubMed (MEDLINE), Science Direct, and Embase was conducted from its inception until February 2024 for randomized controlled trials (RCTs) including patients aged >18 years undergoing cardiac procedures with underlying chronic kidney disease (CKD), estimated glomerular filtration rate (eGFR) 20–60 ml/min/1.73 m^2^ and left ventricular ejection fraction (LVEF) >50%. The outcomes of interest were risk of CIN, risk of renal replacement therapy (RRT), in-hospital mortality and 30-day mortality, major adverse cardiovascular events (MACE), changes in serum creatinine (sCr) levels, and incidence of pulmonary edema. A random-effects meta-analysis was performed using Review Manager (RevMan) [Computer Program] Version 5.4 Cochrane Collaboration.

**Results:**

A total of 9 RCTs including 3,215 patients with CKD undergoing cardiac procedures on volume expansion strategies were included with 1,802 patients on the RG system and 1,413 patients using alternate volume expansion techniques. Pooled analysis of 9 RCTs reported a significantly lower risk of CIN in patients using the RG system vs. control [OR 0.51 (0.35, 0.74), *P* = 0.0004; I^2^ = 55%]. There was no significant difference in the risks of RRT, in-hospital mortality, 30-day MACE, pulmonary edema, or change in sCr levels.

**Conclusion:**

This meta-analysis indicates the beneficial utilization of the RG system in populations with moderate-to-high risk and underlying CKD undergoing cardiac interventions in preventing CIN. However, it did not demonstrate a notable impact on mortality, RRT, MACE, pulmonary edema, and sCr levels when compared to the control group.

## Introduction

Globally, an increasing number of patients undergo cardiac procedures such as coronary angiography and percutaneous coronary intervention (PCI), necessitating the use of contrast media (CM). While CM are generally safe, the occurrence of contrast-induced nephropathy (CIN), also called as contrast-associated acute kidney injury (CA-AKI), remains a common complication following cardiac procedures (1). The reported incidence of acute kidney injury (AKI) varies by procedure, ranging from 10% to 30% after transcatheter aortic valve replacement (TAVR), 13% and 18.9% following PCI, and 1.6%–3.3% after diagnostic interventions (2–4). The rate also varies based on the risk profile of patients, reaching 1%–2% in the general population and up to 50% in certain high-risk patient sub-groups (1). Key predictors of CIN include chronic kidney disease (CKD), diabetes, age, CM volume, and reduced left ventricular ejection fraction (LVEF) (5). The Kidney Disease: Improving Global Outcomes (KDIGO) guidelines define CIN as a ≥25% increase in serum creatine (sCr) or an absolute increase of at least 0.5 mg/dl 48–72 h post-contrast administration not attributable to other causes (5). Notably, this iatrogenic complication stands as the third leading cause of hospital-acquired AKI and is also associated with significantly increased risks of serious short- and long-term adverse clinical outcomes (1, 6–8).

Although there is no pharmaceutical intervention proven to effectively prevent or treat CIN, the KDIGO guidelines for AKI recommend pre-contrast exposure risk assessment and the initiation of supportive measures, including volume management, maintenance of adequate blood pressure using isotonic saline or sodium bicarbonate, administration of pharmaceutical agents like N-acetylcysteine (NAC) and statins, and renal replacement therapies (RRT) such as hemofiltration or hemodialysis (9–11). Avoidance of nephrotoxic drugs and an optimization of CM volume are also advised for at-risk patients (9–11). Implementation of these strategies in high-risk individuals has demonstrated a reduction in the AKI rate from 71.1% to 55.1% (12). Despite these measures, CIN remains a significant source of morbidity and mortality in patients undergoing cardiac interventions (13). The single most effective measure in preventing CIN is peri-procedural intravascular hydration, typically achieved through normal saline infusion at a rate of 1 ml/kg/h (or 0.5 ml/kg/h if LVEF is 35% or New York Heart Association functional class >II) from 12 h before to 24 h after CM exposure (14). However, this approach has limitations in high-risk patients such as the increased risk of acute pulmonary edema, and electrolyte imbalance. Tailored hydration regimens, such as left ventricular end-diastolic pressure (LVEDP)-guided and urine flow rate (UFR)-guided hydration, have been proposed to address these challenges (15–17). Recently, the RenalGuard (RG) system has emerged as a new tool, demonstrating a safer and more efficient hydration, and reducing the incidence of AKI by 60%–75% in several studies (16–24).

The RG system utilizes forced diuresis with low-dose furosemide (0.25–0.5 mg/kg) along with matched rehydration, a strategy endorsed by the European Society of Cardiology (ESC) guidelines for preventing CIN (25). The system components include a closed-loop fluid management system, a high-volume automatic fluid infusion pump, a high-accuracy dual-weight measuring system, a single-use intravenous (I.V.) set, and a urine collection system linked to a standard foley catheter. Notable features include real-time displays of urine and replacement fluid volumes, alerts for draining the urine bag or replacing the hydration fluid bag, and safety features like automatic air and occlusion detection. The system calculates urine flow rate, measures urine volume, and infuses a predetermined hydration fluid volume to match urine output, maintaining extracellular volume. Referred to as “matched hydration”, the RG system enables the users to set parameters for achieving a net fluid gain or loss and allows infusion of a fluid bolus upon user request (22).

So far, the RG system has been tested in a few randomized controlled trials (RCTs) and meta-analyses (16–24, 26–31). However, these analyses are restricted by their small sample sizes and have demonstrated conflicting findings. To enhance statistical power, overcome methodological limitations, and incorporate the latest evidence, we conducted this meta-analysis on the effectiveness of the RG system in preventing CIN in patients undergoing cardiac interventions.

## Methods

This meta-analysis is conducted in line with the Preferred Reporting Items for Systematic Review and Meta-Analysis (PRISMA) guidelines and follows the framework laid out by the Cochrane Collaboration (32).

### Data sources and search strategy

A comprehensive literature search of PubMed, Science Direct and Embase was conducted from its inception until February 2024. There were no filters applied based on time, language, geographical location, author name and year of publication. The search strategy used in the electronic databases included keywords such as “Contrast-Induced Nephropathy”, “Contrast-Induced Renal Damage” “RenalGuard Therapy” and “Chronic Kidney Disease Patients”. A detailed description of the search strategy is given in Supplementary Table S1.

### Study selection

Articles retrieved from the literature search were exported to Endnote Reference Library (Version X7.5; Clarivate Analytics, Philadelphia, Pennsylvania) software, and the duplicates were identified and removed. The remaining articles were then thoroughly reviewed by independent reviewers (FY and HN), ensuring that the selected articles met the predefined eligibility criteria.

The eligibility criteria for this meta-analysis included patients (a) aged >18 years with underlying chronic kidney disease (CKD) (b) undergoing cardiac procedures and at risk of developing AKI, estimated glomerular filtration rate (eGFR) 20–60 ml/min/1.73 m^2^, and LVEF >50%. Studies were selected if they were RCTs and reported the following outcomes: risk of CIN, risk of RRT, in-hospital mortality and 30-day mortality, major adverse cardiovascular events (MACE), changes in sCr levels, and incidence of pulmonary edema.

### Data extraction and quality assessment

Data was extracted and verified by two independent reviewers (FY and H.N). Data extracted from each study included: study design, publication year, study population, sample size, number of patients in each group (use of RG vs. control), general patient characteristics (age and sex), pre-operative eGFR, comorbidities, and the endpoints of the study. The primary outcome included the risk of CIN. Secondary outcomes comprised risks of RRT, in-hospital mortality, 30-day mortality, MACE, changes in sCr levels and incidence of pulmonary edema. Quality assessment was conducted via the Cochrane Risk of Bias (RoB2) tool to determine the quality of the included RCTs (33). Any discrepancy was resolved by consensus and discussion.

### Statistical analysis

The analysis was performed using Review Manager (RevMan) [Computer Program] Version 5.4 Cochrane Collaboration. A random-effects model with Mantel-Haenszel weighting was used to estimate the overall odds ratio (OR) and 95% confidence intervals (CIs) for variables with dichotomous outcomes. The inverse variance was used to pool continuous outcomes as mean and standard deviation (SD) with 95% confidence intervals (CIs). Outcomes reporting median and interquartile ranges were converted to mean and standard deviation using methods by Wan et al. and Luo et al. (34, 35). Each outcome is represented by a forest plot. Heterogeneity across the pooled studies was assessed using the Higgins I^2^ statistics. An I^2^ value of 25%–50% was considered mild, 50%–75% as moderate, and >75% as severe heterogeneity (36). Outcomes with studies that reported a high % of heterogeneity were subjected to sensitivity analysis to explore the effect of each study on the pooled estimate. A funnel plot could not be obtained to detect publication bias as each outcome had less than ten studies. A *p*-value <0.05 was considered significant throughout the study.

## Results

### Study selection and baseline characteristics

**Figure 1:**
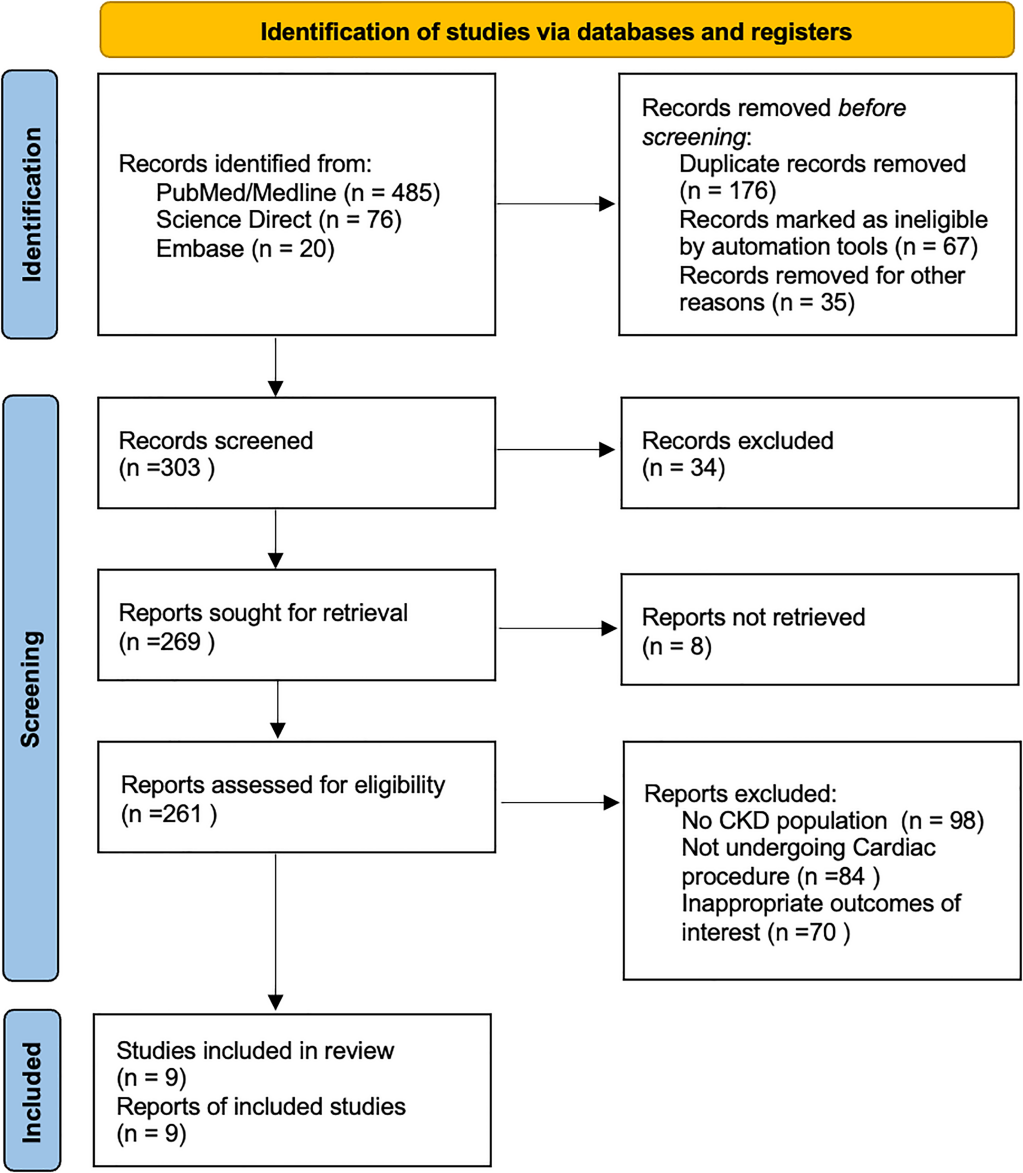
PRISMA flow chart.

The initial literature search yielded 581 potential articles, of which 261 were assessed for detailed evaluation, as shown in Figure 1. A total of 9 RCTs were shortlisted for inclusion in the quantitative and qualitative synthesis after removing studies not fulfilling the eligibility criteria (16–24).

Meta-analysis was performed on a total population of 3,215 patients with CKD undergoing cardiac procedures on volume expansion strategies. Of these techniques, the RG system was used on 1,802 patients and 1,413 patients used alternate volume expansion techniques. The characteristics of all included studies are presented in Table 1.

**Table 1:** Baseline characteristics.

Trial/author, year	Total	RG (*n*)	Control (*n*)	Age (years) ± SD or (IQR)		Male gender, *n* (%)		Diabetic, *n* (%)		CABG, *n* (%)		Left ventricular ejection fraction, %		CAD (%)		Hypertension (%)	
				RG	Control	RG	Control	RG	Control	RG	Control	RG	Control	RG	Control	RG	Control
Witwer S, 2022: STRENGTH	248	122	126	79.1 ± 8.8	78.5 ± 9.6	62 (50.8)	63 (50)	60 (49.2)	50 (39.7)					66 (54.1)	68 (54.0)	101 (82.8)	103 (81.7
J. Mirza, 2022: CINEMA	1,205	799	406	62.3 ± 7.5	65.5 ± 8.2	457 (57.2%)	241 (59.4%)	451 (56.4%)	192 (47.3%)			51.17 ± 9.53	52.02 ± 9.94			587 (73.5%)	234 (57.6%)
Luckraz, 2021: KIDNEY	220	110	110	67.8 (9.3)	67.0 (9.2)	87 (79)	84 (76)	80 (72.7)	80 (72.7)	53 (48)	59 (54)						
Briguori, 2020	708	355	353	74 ± 8	74 ± 8	233 (65.5)	207 (59.0)	177 (50.0)	175 (49.5)			49 ± 10	50 ± 11	71 (20.0)	74 (21.0)	323 (91.0)	321 (91.0)
Arbel, 2019: REDUCE-AKI	136	68	68	84.2 (80.8– 87)	84.5 (81.2–87.1)	38 (56%)	42 (62%)	26 (38%)	28 (41%)	9 (13%)	8 (12%)			36 (53%)	34 (50%)	62 (91%)	59 (87%)
Usmiani, 2016: AKIGUARD	124	59	65	76 ± 9	75 ± 8	46 (79%)	46 (71%)	22 (37%)	22 (34%)	7 (12%)	12 (18%)	53 ± 10	50 ± 14			49 (83%)	55 (85%)
Barbanti, 2015: PROJECT TAVI	112	56	56	82 (78–83)	81 (78–84)	22 (39.3)	33 (58.9)	16 (28.6)	22 (39.3)	3 (5.4)	6 (10.7)	53.6 ± 13	55.5 ± 8.7			42 (75.0)	49 (87.5)
Marenzi, 2012: MYTHOS	170	87	83	73 ± 7	74 ± 8	68 (78%)	65 (78%)	38 (44%)	29 (35%)	28 (32%)	21 (25%)	51 ± 13	52 ± 13			72 (83%)	69 (83%)
Briguori, 2011: REMEDIAL	292	146	146	75 ± 9	76 ± 8	103 (70.5)	88 (60.5)	104 (71)	101 (69)			48 ± 10	46 ± 11	27 (18.5)	28 (19)	144 (98)	143 (98)

## Primary outcome

### Risk of CIN

**Figure 2:**
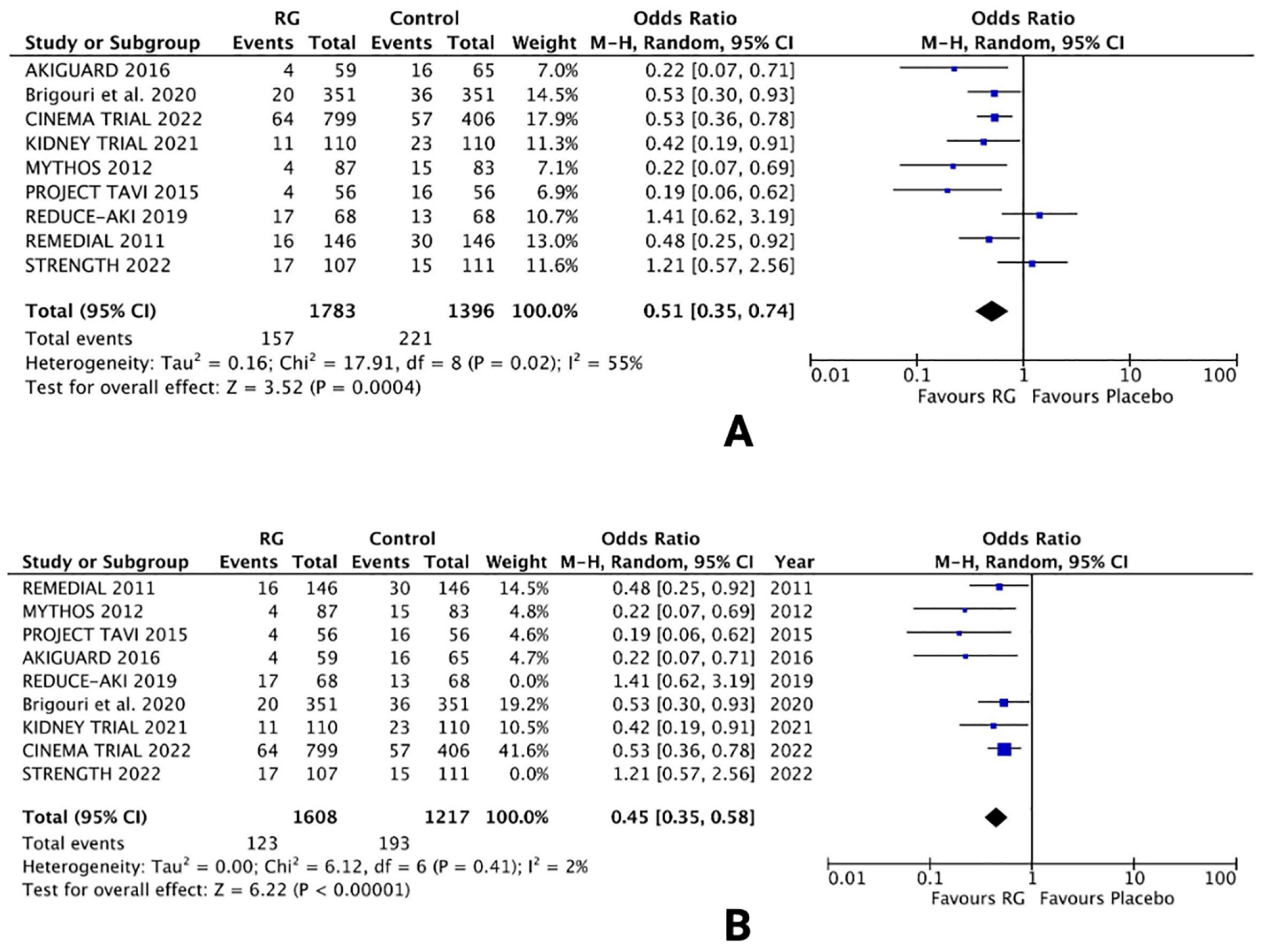
Forest plot of **(A)** risk of CIN **(B)** sensitivity analysis of risk of CIN in CKD patients using renalGuard device versus control. Blue squares and their corresponding lines are the point estimates and 95% confidence intervals per each study. Black diamonds represent the pooled effect estimate.

All 9 trials assessed the risk of CIN, defined as post-procedural AKI following the treatment options (16–24). An overall pooled analysis reported a significantly lower risk of CIN in patients using the RG system vs. control [OR 0.51; 95% CI (0.35, 0.74), *P* = 0.0004; I^2^ = 55% Figure 2A].

Owing to the moderate heterogeneity in the results, RCTs with low-quality assessment scores, varying demographics and number of patients were excluded in a sensitivity analysis. Removing 2 RCTs [REDUCE-AKI 2019 (20) and STRENGTH 2022 (24)] lowered the heterogeneity from I^2^ = 55% to I^2^ = 2%. The results were unchanged, with the RG system carrying a lower risk of CIN vs. control [OR 0.45; 95% CI (0.35, 0.58), *P* < 0.0001; Figure 2B], respectively.

## Secondary outcomes

### Risk of RRT

**Figure 3:**
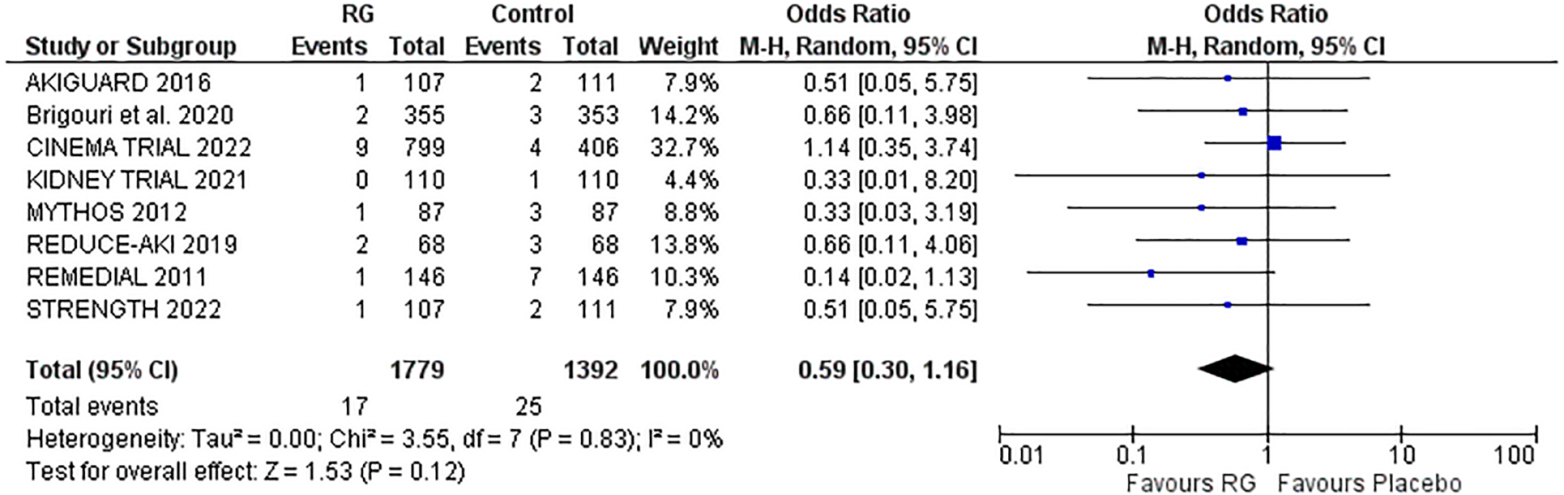
Forest plot of risk of RRT in CKD patients using RenalGuard device versus control. Blue squares and their corresponding lines are the point estimates and 95% confidence intervals per each study. Black diamonds represent the pooled effect estimate.

Eight trials reported the risk of RRT following the treatment options (16, 17, 19–24). A random effects analysis yielded no significant difference in the risk of RRT with RG device vs. control [OR 0.59; 95% CI (0.30, 1.16), *P* = 0.12; I^2^ = 0% Figure 3].

### In-hospital mortality

**Figure 4:**
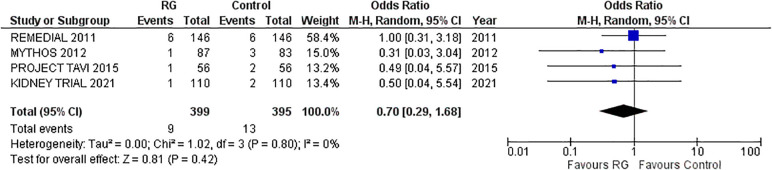
Forest plot of in-hospital mortality in CKD patients using RenalGuard device versus control. Blue squares and their corresponding lines are the point estimates and 95% confidence intervals per each study. Black diamonds represent the pooled effect estimate.

Four studies reporting in-hospital mortality following the procedure were pooled via a random-effects model (16–18, 22). The incidence of in-hospital mortality was not significantly different between the two groups, RG system vs. control [OR 0.70; 95% CI (0.29, 1.68), *P* = 0.80; I^2^ = 0% Figure 4].

### 30-day MACE

**Figure 5:**
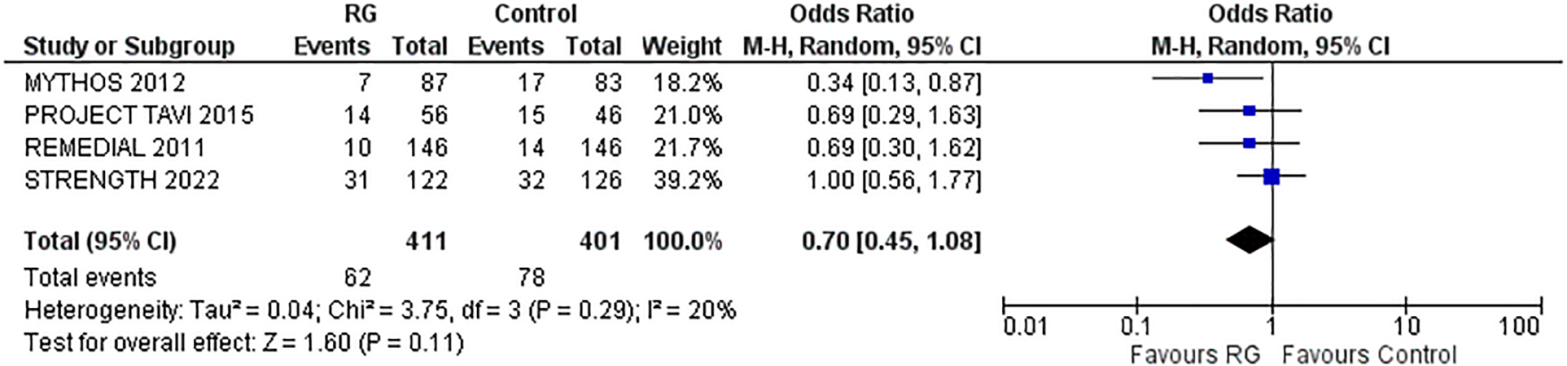
Forest plot of 30-day MACE in CKD patients using RenalGuard device versus control. Blue squares and their corresponding lines are the point estimates and 95% confidence intervals per each study. Black diamonds represent the pooled effect estimate.

A total of four studies reporting 30-days MACE were analyzed by pooling a random-effects model (16–18, 24). The results yielded a non-significant difference in CKD patients undergoing volume expansion strategies with the RG system vs. control [OR 0.70; 95% CI (0.45, 1.08), *P* = 0.11; I^2^ = 20% Figure 5].

### Pulmonary edema

**Figure 6:**
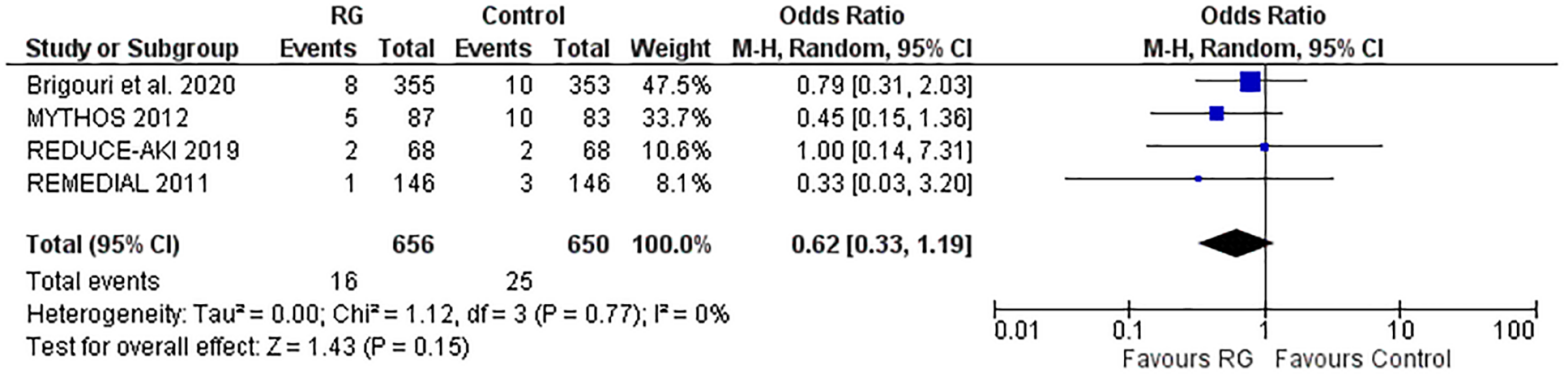
Forest plot of pulmonary edema in CKD patients using RenalGuard device versus control. Blue squares and their corresponding lines are the point estimates and 95% confidence intervals per each study. Black diamonds represent the pooled effect estimate.

Four studies reporting the incidence of pulmonary edema were pooled using a random-effects model (16, 17, 20, 21) and did not yield a significant difference between the RG system vs. the control group [OR 0.62; 95% CI (0.33, 1.19), *P* = 0.15; I^2^ = 0% Figure 6].

### Change in serum creatinine level

**Figure 7:**
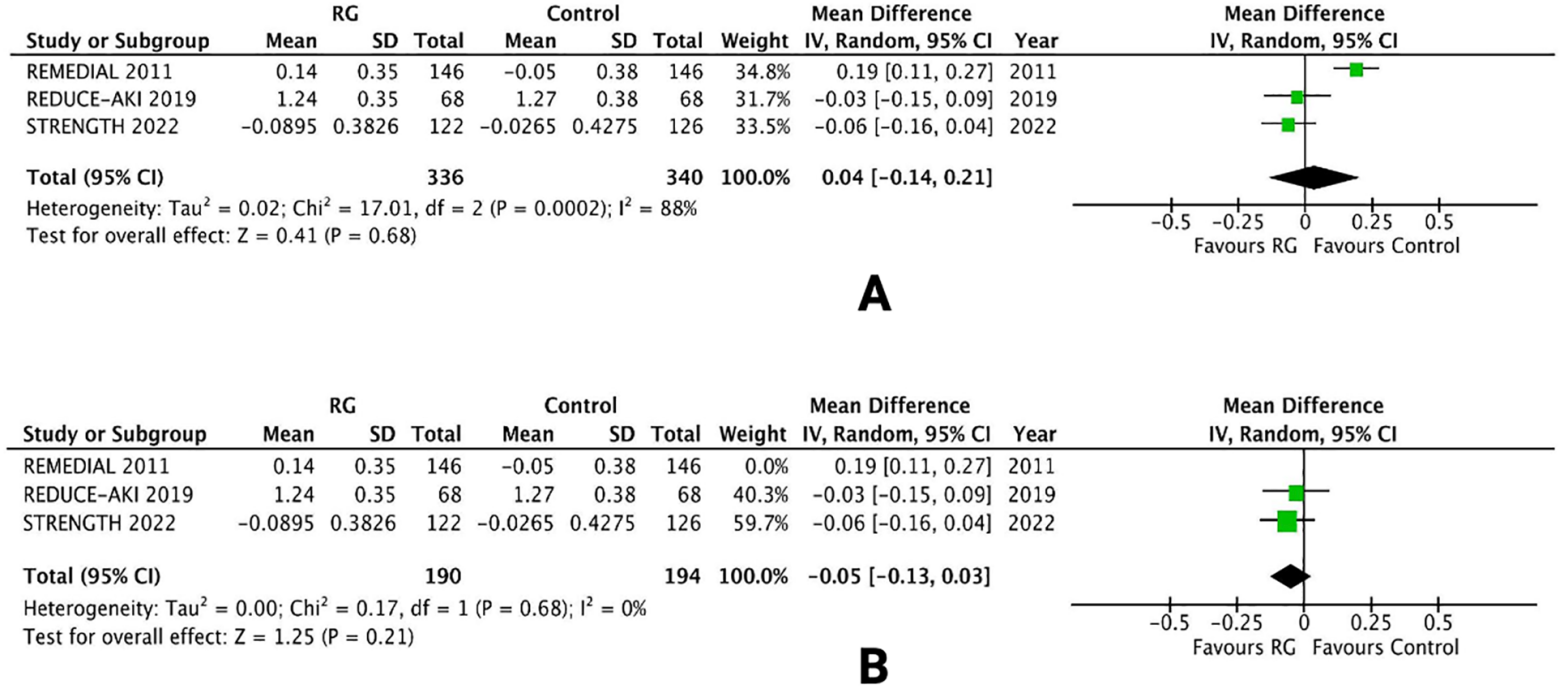
Forest plot of **(A)** change in sCr levels **(B)** leave-one-out sensitivity analysis in CKD patients using renalGuard device versus control. Blue squares and their corresponding lines are the point estimates and 95% confidence intervals per each study. Black diamonds represent the pooled effect estimate.

Three RCTs reported changes in the sCr level from baseline, following treatment protocols (16, 20, 24). Mean values and standard deviation pooled via inverse variance did not conclude a difference between patients undergoing volume expansion with RG system vs. control [WMD 0.04; 95% CI (−0.14, 0.21), *P* = 0.68; I^2^ = 88% Figure 7A].

A leave-one-out sensitivity analysis was performed to reduce the heterogeneity of the outcome. Removing one trial [REMEDIAL 2011 (16)] reduced the heterogeneity from I^2^ = 88% to I^2^ = 0%. There was no change in the overall results and the difference between the two groups was not significant [WMD −0.05; 95% CI (−0.13, 0.03), *P* = 0.21; Figure 7B].

### Quality assessment and risk of bias

The Cochrane RoB2 Tool was used to carry out the methodological quality assessment of the nine RCTs determined a “low” risk of bias overall. All domains reported a low risk of bias for the RCTs as the procedure, analysis, and outcomes were adequate in the study. A detailed quality assessment is shown in Supplementary Figure S2.

## Discussion

In this pooled meta-analysis of RCTs, the RG system, employing furosemide-induced forced diuresis with matched hydration, demonstrated a significant reduction in the risk of CIN in CKD patients undergoing cardiac interventions compared to control strategies. However, no significant impact was observed on the need for RRT, 30-day MACE, in-hospital mortality, pulmonary edema, or sCr levels when compared to the control group. These findings underscore the RG system's potential in mitigating AKI during contrast-intensive cardiovascular procedures.

Even in its milder forms, CKD independently elevates the risk of coronary artery disease, affecting nearly one-third of acute coronary syndromes (ACS) patients and contributing to increased cardiovascular morbidity and mortality (37). Cardiac interventions, including coronary angiography and PCI, pose additional challenges in CKD patients due to the heightened risk of CIN (38). Comprehensive evaluation using conventional risk scores, and preventive therapies like pre-hydration are recommended before CM administration but concerns about volume overload often lead to suboptimal hydration (4, 39). The optimal treatment for preventing CIN remains undefined, with the use of diverse interventions such as anti-inflammatory agents including statins, vasodilators including dopamine, fenoldopam, captopril, prostacyclin analogs and endothelin antagonists, diuretics, adenosine antagonists, antioxidants like NAC, ascorbic acid, and sodium bicarbonate, anti-ischemic agents (trimetazidine), and RRT showing inconsistent results. Notably, hydration and minimizing CM amount are the only strategies conclusively demonstrated to reduce CIN risk (26).

In alignment with previous studies, our analysis affirms that the RG system is a secure and effective approach for periprocedural hydration, notably reducing the incidence of CIN in high-risk patients with underlying CKD undergoing cardiac interventions (26–31). CM exerts direct toxicity on renal tubular cells, inducing vacuolization, altered mitochondrial function, and apoptosis. Additionally, CM disrupts the balance between oxygen delivery and consumption in the renal medulla, leading to reactive oxygen species (ROS) production, increased osmotic pressure, and viscosity slowing the renal blood flow. Endothelial damage in the vasa recta reduces nitric oxide and prostaglandin production which are necessary for vasodilation, thus exacerbating medullary ischemia (40). While the exact mechanism of the RG system remains unclear, it likely operates through a dual strategy: diuretic-forced diuresis with tailored fluid administration, promoting high urine output (>300 ml/h) for CM dilution and elimination, and volume expansion inhibiting various physiological pathways thus, enhancing the renal medulla perfusion, and minimizing exposure time to CM-induced toxicity (22). Notably, in some of the included trials, the RG group received a larger volume of intravenous hydration (2,598 vs. 1,709 ml), potentially contributing to the observed lower CIN rates in the RG group (21).

In contrast to previous meta-analyses demonstrating a significant reduction in the need for RRT (hemodialysis, hemofiltration, etc.) with the RG system, our meta-analysis found similar rates of RRT between RG and alternative volume expansion technique recipients (26–29). This discrepancy may be attributed to the heterogeneity in treatment strategies in the control group and a low number of events in previous analyses (26–29). Additionally, the inclusion of observational studies and fewer RCTs in previous meta-analyses might have overestimated the effect size (26–28). The observed similarity in RRT need despite lower CIN rates in RG recipients could be influenced by factors beyond CIN prevention. Patients undergoing contrast-related procedures may have pre-existing renal conditions, varied baseline renal function, and individual responsiveness to renal protection strategies, that elevate their overall risk of requiring RRT, irrespective of CIN prevention efforts. The multifactorial nature of renal complications in patients undergoing medical procedures makes it challenging to attribute the necessity for RRT solely to the prevention or occurrence of CIN. Moreover, variability in study designs, procedural factors (e.g., general anesthesia, blood pressure, transapical access, bleeding, and rapid pacing), post-procedural (e.g., blood transfusion) and follow-up protocols, and type of procedures (e.g., emergent vs. elective, complex interventional vs. diagnostic) across included studies could contribute to the varying effectiveness of RG in preventing renal complications, explaining the lack of significant statistical difference in RRT need in our meta-analysis (16–24).

Crucially, the RG system demonstrated benefits without compromising safety in our meta-analysis. Consistent with prior research by Putzu et al. and Occhipinti et al., we found no significant impact on in-hospital mortality and 30-day MACE between the two study arms (27, 30). However, this differs from previous meta-analyses by Mattathil et al. and Shah et al., which reported a 59% decrease in MACE (26, 28). Variable treatment regimens in the control group, such as NAC, sodium bicarbonate, saline and statins, might explain these findings (9, 16–21, 23, 24). NAC's antioxidant properties and sodium bicarbonate's buffering capacity might account for the lower rates of adverse outcomes in the control arm (11). Differences in tubule-glomerular feedback activation due to diverse sodium salt solutions may also play a role (41). The RG group's lower, though not significant, rates of post-operative ACS and stroke suggest potential protective effects at cerebral and cardiac levels. Further research is needed to confirm these hypotheses.

Considering the lower CIN rate, this could also positively impact the economy, given the high costs associated with AKI. National Health Service Kidney Care estimated the annual cost of AKI to be $700 million to $1 billion, exceeding the combined expenditures on breast cancer, lung cancer, and skin cancer (42). Longer hospitalization time due to complications related to CIN-induced AKI appeared to be the major driver for this increased economic burden (42). Therefore, a detailed assessment of the impact of RG therapy on economic outcomes warrants further investigation.

A noteworthy aspect of RG's safety is its ability to prevent issues related to unbalanced hydration. In line with the study by Putzu et al., our meta-analysis revealed a similar safety profile, with non-significant differences in rates of pulmonary edema compared to the control (27). The closed-loop fluid management system of RG is designed to measure urine output and replace it in real-time with an equal volume of intravenously infused saline. This precisely matched fluid replacement aims to minimize the risk of over- or underhydration and maintain euvolemia (22). The unexpected finding of a protective, yet non-significant association between the RG system and acute pulmonary edema has notable implications for patient management and prognosis. The beneficial effect may be linked to the use of furosemide, blocking tubular sodium reabsorption and decreasing tubular workload (30). Despite the higher volume of periprocedural hydration in the RG group, similar rates of pulmonary edema compared to the control group strengthen the safety profile of this system (21).

Currently, there is a lack of standardized guidelines for hydration protocols, with patients commonly receiving overnight intravenous hydration before and after procedures. Studies have demonstrated comparable outcomes in preventing CIN with hydration administered 1 h before and 6 h after the procedure, especially in outpatient settings. This suggests that the infusion rate may be a crucial factor, emphasizing the potential benefits of quicker hydration over a shorter duration (15, 43). These findings further underscore the safety and efficacy of RG in preventing overhydration-related complications during cardiac interventions.

Our meta-analysis surprisingly did not show a significant disparity in sCr levels between the study groups. The RG system's forced diuresis, coupled with matched intravascular hydration, effectively not only guards against CIN but also swiftly eliminates excess creatinine and nitrogenous wastes from the bloodstream, akin to standard hydration regimens. This efficient washout process aids in maintaining sCr within a range comparable to standard regimens, preventing prolonged kidney exposure to contrast materials. The rapid washout mechanism also contributes to stable hemodynamics and renal perfusion, averting excessive fluctuations in sCr (22). Additionally, the lower baseline sCr levels (1–2 mg/dl) among the included participants might have contributed to the diminished protective effects of the RG system, potentially explaining the lack of statistical significance (16–24). Furthermore, concurrent interventions or medications in both groups might also have influenced sCr levels.

### Strengths and limitations

This meta-analysis represents a comprehensive and up-to-date investigation on the topic, incorporating all the RCTs available to date to provide a thorough evaluation of the RG system's safety and efficacy. Importantly, a detailed systematic review of several databases was carried out to minimize the risk of overlooking relevant publications. Despite the suggestion of tailoring hydration regimens as an alternative, supporting evidence for the RenalGuard system's efficacy remains limited, with a low-grade level of evidence due to the variability in patient inclusion criteria across individual trials and observational registries (16–24, 44, 45). Notably, our meta-analysis overcomes those limitations as it directly compares the RG system with standard hydration strategies, emphasizing the inclusion of the most recent evidence, avoiding the heterogeneity introduced by indirect comparisons, and utilizing random-effects models to mitigate between-trial variability. The exclusive incorporation of RCTs only further enhances the overall quality of evidence compared to previous meta-analyses.

However, our meta-analysis has some limitations that need to be acknowledged. The identification of only nine studies related to RG usage in the literature search suggests that a greater number of RCTs would have strengthened the analysis (16–24). Most of these studies were single center with small sample sizes, potentially impacting the generalizability of findings. The absence of individual participant data necessitated a study-level statistical approach, and the variations in study protocols, follow-up intervals, lack of standardization in CIN definitions across the included trials, and pre-procedural hydration strategies in the control group might have introduced heterogeneity. Four (16–18, 21) out of nine studies used the Mehran score (4) to predict the risk of CIN post-procedure and therefore, could not be used to stratify the study's patient population. However, sensitivity analyses confirmed consistent directions of effects. The application of RG was predominantly in non-emergent coronary and TAVR procedures, raising uncertainties about its efficacy in more urgent scenarios. The external validity of results is confined to patients with CKD or those at high risk, emphasizing the importance of precise risk stratification for optimal outcomes and cost reduction.

Despite the positive outcomes in the meta-analysis, limitations in RG technology need to be considered. An observational study suggested a peri-procedural UFR >450 ml/h for effective CIN prevention with RG, whereas the studies analyzed in our meta-analysis targeted >300 ml/h (16–24, 45). The requirement for a Foley catheter for real-time monitoring restricts RG use in patients unable to have urinary catheters (46). The ideal diuretic dosage, UFR, and potential urological complications related to the Foley catheter need further exploration to optimize this system. While cost-effectiveness is appealing, the RG system's potential high cost prompts the need for more cost-efficient alternatives (30, 46). Additionally, the studies included in the analysis lack data to analyze the impact of contrast media osmolality and evaluate renal safety post-procedure.

### Prospects

This meta-analysis on the RG system's efficacy in preventing CIN provides valuable insights that can inform healthcare guidelines and policies, promoting evidence-based practices for improved patient outcomes in contrast-intensive cardiovascular procedures. Our findings support the inclusion of the RG system as a recommended strategy for periprocedural hydration in high-risk patients undergoing cardiac procedures, especially those with CKD. Hospitals and healthcare facilities may consider incorporating the system into standard protocols to mitigate the risk of CIN. The study's observations on economic impact and safety profiles could influence health policies, emphasizing the potential cost savings and safety benefits associated with the RG system. Furthermore, the research suggests a need for refined hydration protocols, focusing on infusion rates and individualized patient factors to enhance CIN prevention strategies. Additionally, in the upcoming period, it is essential to conduct new trials, specifically comparing the effectiveness of furosemide with matched hydration, both with and without the RG system. These trials should aim to clarify the potential efficacy of the RG system not only in cardiac interventions but also in other medical procedures like diagnostic radiology, endovascular procedures, and potentially during treatments involving nephrotoxic chemotherapeutic agents such as cisplatin and methotrexate.

## Conclusion

Our meta-analysis indicates that the utilization of the RG system in populations with moderate-to-high risk and underlying CKD undergoing cardiac interventions is effective in preventing CIN. However, it did not demonstrate a notable impact on in-hospital mortality, progression to RRT, short-term MACE, pulmonary edema, and sCr levels when compared to the control group. Consequently, there is a need for large-scale, adequately powered, RCTs to establish the benefits of the RG system in improving clinically significant endpoints and to endorse its widespread implementation.

## Data Availability

The original contributions presented in the study are included in the article/Supplementary Material, further inquiries can be directed to the corresponding author.
