# Supplementary material for: Efficacy of the Renal-guard system in the prevention of contrast-induced nephropathy following cardiac interventions among patients with chronic kidney disease
Source: Front Cardiovasc Med. 2025 Mar 3;12:1438076. doi: 10.3389/fcvm.2025.1438076 (PMC11911345; doi:10.3389/fcvm.2025.1438076)
Supplement: Supplementary file 1 [file Datasheet1.docx]

**ONLINE SUPPLEMENTARY**

**Supplementary Table 1. Search Strategy**

| **PubMed/Medline *(485)*** | (("Contrast-Induced"[All Fields] AND ("kidney diseases"[MeSH Terms] OR ("kidney"[All Fields] AND "diseases"[All Fields]) OR "kidney diseases"[All Fields] OR "nephropathies"[All Fields] OR "nephropathy"[All Fields])) OR ("Contrast-Induced"[All Fields] AND ("renal"[All Fields] OR "renals"[All Fields]) AND ("damage"[All Fields] OR "damaged"[All Fields] OR "damages"[All Fields] OR "damaging"[All Fields])) OR ("renal insufficiency, chronic"[MeSH Terms] OR ("renal"[All Fields] AND "insufficiency"[All Fields] AND "chronic"[All Fields]) OR "chronic renal insufficiency"[All Fields] OR ("chronic"[All Fields] AND "kidney"[All Fields] AND "disease"[All Fields]) OR "chronic kidney disease"[All Fields])) AND (("RenalGuard"[All Fields] AND ("system"[All Fields] OR "system s"[All Fields] OR "systems"[All Fields])) OR ("RG"[All Fields] AND ("system"[All Fields] OR "system s"[All Fields] OR "systems"[All Fields])) OR (("volum"[All Fields] OR "volume"[All Fields] OR "volumes"[All Fields] OR "voluming"[All Fields]) AND ("expanse"[All Fields] OR "expanses"[All Fields] OR "expansion"[All Fields] OR "expansions"[All Fields] OR "expansive"[All Fields] OR "expansively"[All Fields]))) |
| --- | --- |
| **Science Direct *(76)*** | (Contrast-Induced Nephropathy OR Contrast-Induced Renal Damage) AND (RenalGuard System) |
| **Embase (20)** | (Contrast-Induced Nephropathy OR Contrast-Induced Renal Damage) AND (RenalGuard System) |

**Supplementary Figure 2. Quality Assessment of RCTs via ROB2**

**
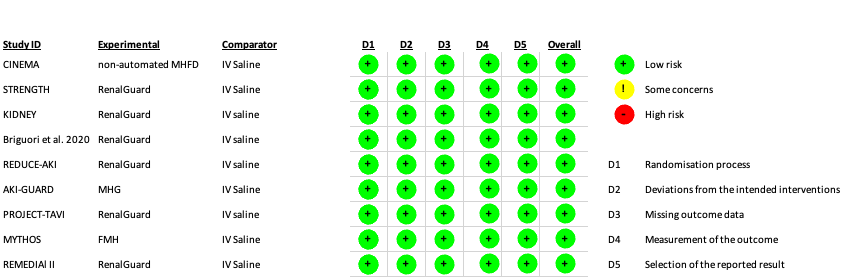
**
